# Determinants of cervical cancer screening among women living with HIV in Lesotho using nationally representative 2023/24 DHS data

**DOI:** 10.1038/s41598-026-37180-z

**Published:** 2026-01-28

**Authors:** Tseganesh Asefa, Hiwot Tezera Endale, Tiget Ayelgn Mengstie, Mihret Getnet

**Affiliations:** 1 Clinical Trial Directorate, Armauer Hansen Research Institute, Addis Ababa, Ethiopia; 2 Department of Medical Biochemistry, School of Medicine, College of Medicine and Health Sciences, University of Gondar, Gondar, Ethiopia; 3 Department of Epidemiology and Biostatistics, Institute of Public Health, College of Medicine and Health Sciences, University of Gondar, Gondar, Ethiopia; 4 Department of Human Physiology, School of Medicine, College of Medicine and Health Sciences, University of Gondar, Gondar, Ethiopia

**Keywords:** Cervical cancer, Determinants, Human immunodeficiency virus, Lesotho, Screening, Cancer, Health care, Risk factors

## Abstract

Women living with HIV are more prone to develop cervical cancer since they have a compromised immune system; hence, they need to be screened continuously in an attempt to identify and prevent it. Despite Lesotho’s high HIV prevalence (25.6%), cervical cancer screening coverage and its determinants among women living with HIV remain insufficiently characterized. This study aimed to quantify the rate and determinants of cervical cancer screening among women living with HIV using the 2023/24 Lesotho DHS data. Cross-sectional analysis was performed using the Lesotho DHS Individual Women’s Recode file. A weighted sample of 611 HIV-positive women aged 25 years and older participated in the study, as this age group is eligible for cervical cancer screening. Individual and community-level factors were determined using multilevel mixed-effects logistic regression. The level of significance was determined by the 95% confidence interval and a *p* value less than 0.05 for associations. The total prevalence of cervical cancer screening among women living with HIV was 85.4%. Women aged 40–44 years (adjusted odds ratio [AOR] 4.14; 95% confidence interval [CI] 1.53–11.18) and those who had a clinical breast exam (AOR 5.53; 95% CI 2.54–12.05) were more likely to undergo cervical cancer screening, whereas low parity (AOR 0.19; 95% CI 0.05–0.78) and rural residence (AOR 0.50; 95% CI 0.25–0.99) were associated with lower odds of screening. Adoption of cervical cancer screening among women living with HIV in Lesotho is high, with most screened women receiving normal results. Screening uptake varied by demographics, being higher among older women and those who had breast examinations, while lower among women with low parity and rural residents. Integration of breast and cervical cancer screening, rural outreach targeting, and health education for low-parity women can increase coverage and equity.

## Introduction

Cervical cancer is still one of the most common causes of morbidity and mortality in women worldwide^1,2^. According to GLOBOCAN, in 2022 alone, there were an expected 662,044 new cases and 348,709 fatalities globally; disproportionately, around 90% of the incidence happened in low- and middle-income countries (LMICs)^3,4^. Women living with HIV (WLHIV) are particularly at high risk, experiencing an estimated sixfold risk of acquiring cervical cancer^5^ because HIV infection greatly weakens the immune response; thus, it becomes harder for the body to resist human papillomavirus (HPV) infections that lead to cervical cancer^5,6^. Hence, WLHIV should regularly and timely be screened for cervical cancer to allow for early diagnosis and prevent the disease from progressing. Despite the availability of effective screening technologies, cervical cancer screening coverage in Africa ranges from 13 to 19%^7,8^.

Lesotho, a country with one of the highest HIV prevalence rates globally, estimated at 25.6% among adults^9^, and cervical cancer represents the most common cancer among women in the country. National cervical cancer screening in Lesotho mainly uses visual inspection with acetic acid (VIA), supplemented by cytology and HPV testing^10^. Despite the availability of national cervical cancer screening guidelines, evidence showed that screening coverage in Lesotho remains low, with only 38.9% of women aged 15–49 years having ever been screened for cervical cancer^11^. This coverage is far below the World Health Organization (WHO) target of 70% screening coverage, set as part of the global strategy for cervical cancer elimination^12^. Although national policies in Lesotho recommend initiating cervical cancer screening at age 25 years for women living with HIV^13^, progress toward achieving high screening coverage has been constrained by a lack of integrated services and limited system resources. Beyond structural constraints, sociocultural factors—including gender-related barriers, concerns about privacy, fear of a cancer diagnosis, and stigma associated with screening—continue to hinder utilization of cervical cancer screening services^8,14,15^.

While several studies have examined cervical cancer screening in the general female population in Lesotho, there is limited nationally representative evidence focusing specifically on women living with HIV and accounting for both individual- and community-level determinants of screening utilization. Thus, this study addresses an important issue by using nationally representative and anonymized data from the 2023/24 Lesotho Demographic and Health Survey to assess cervical cancer screening prevalence and determinants among women living with HIV in Lesotho, providing timely evidence relevant to current HIV and cancer prevention strategies.

## Methods and materials

### Study design, period, and setting

This study is a secondary analysis of data from the Lesotho Demographic and Health Survey (DHS), a nationally representative household study conducted in all districts of Lesotho. The DHS data were collected from 27 November 2023 to 29 February 2024 through a cross-sectional survey and were analyzed in 2025, as it was the most recent dataset available at the time of the study.

Lesotho is a small, landlocked country in Southern Africa, covering approximately 30,355 km^2^, the only nation globally where the entire territory exceeds 1,000 m above sea level. With a population of 2.3 million, the population is relatively young, with a median age in the early twenties, and around one‑third resides in urban areas. The country faces socioeconomic challenges and significant health issues, particularly a high HIV prevalence.

### Data source and sampling procedure

Lesotho DHS employed a stratified two-stage cluster sampling design. At the first stage, the country was stratified by district and residence, yielding 29 sampling strata. Within each stratum, enumeration areas (EAs) were selected by probability proportional to size. At stage two, households were selected within each selected EA using systematic random sampling. Sampling weights (v005/1,000,000) were applied to account for oversampling and unequal selection probabilities. Weighted frequencies and percentages were calculated by multiplying each participant’s data by their sampling weight, ensuring that the estimates accurately reflect the national population and provide unbiased, population-representative results.

This study analyzed the Individual Recode (IR) dataset, which includes women aged 15–49 years. Of the 6413 households initially screened, 6,085 women reported having ever been tested for HIV. Women who had not been tested for HIV (n = 328) were excluded. Among those tested, 6,043 received their HIV test results, and 1,285 were identified as HIV-positive.

Women were included in the analysis if they were aged 25–49 years, HIV-positive, and had complete information on cervical cancer screening. Women aged under 25 years (n = 121) were excluded, as they were not eligible for cervical cancer screening according to Lesotho’s national guidelines, which recommend screening for women living with HIV starting at age 25 or upon becoming sexually active^16^. Participants with missing data on the outcome variable or key explanatory variables were also excluded.

**Fig. 1:**
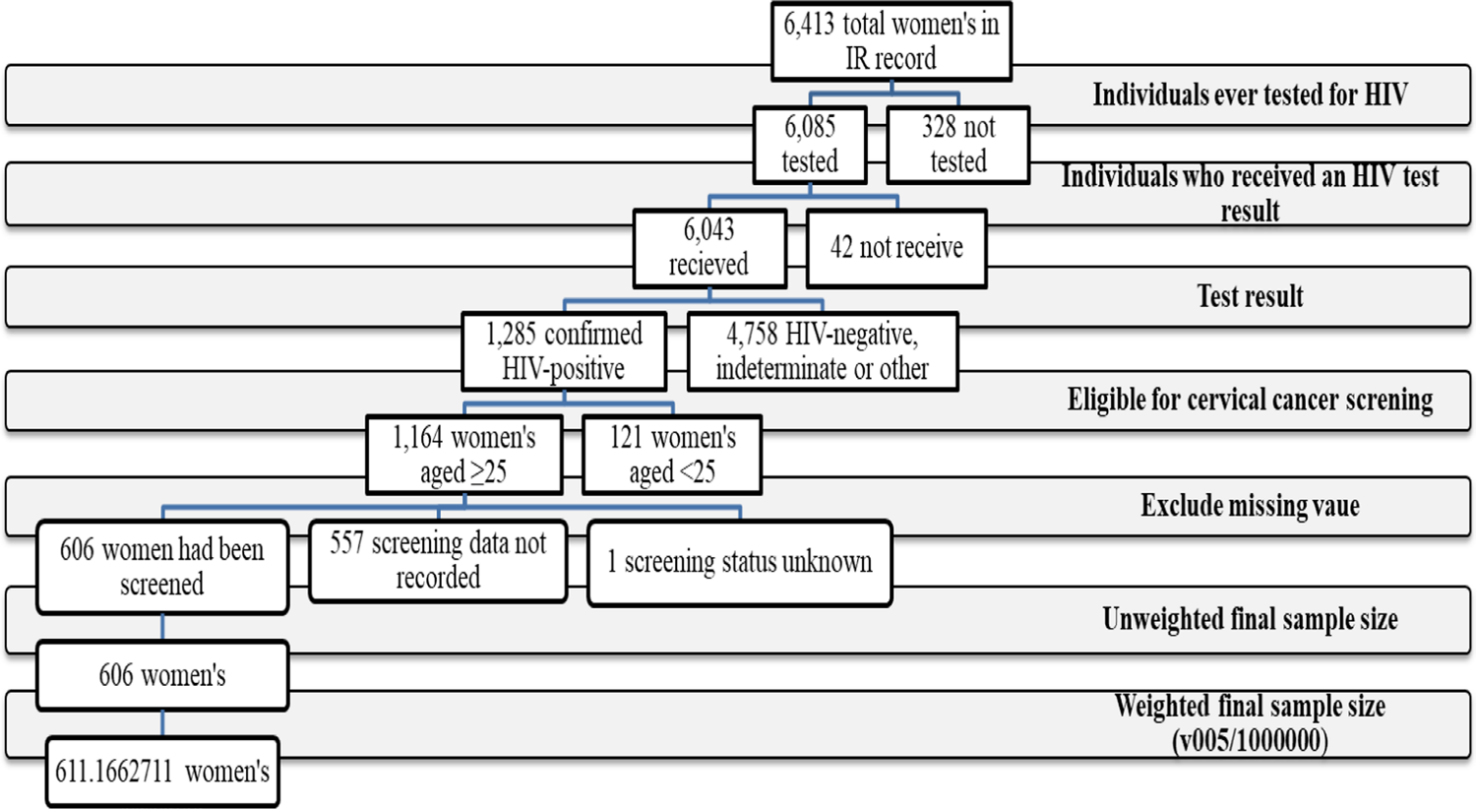
Diagrammatic representation of sample selection in the study.

After applying these criteria, 1,164 women living with HIV were retained, of whom 606 reported having undergone cervical cancer screening. These 606 women constitute the study’s primary sample of interest. Following the application of DHS sampling weights, the final weighted sample size was 611.17 women (Fig. 1).

### Study population

Women aged 25–49 years living with HIV who participated in the 2023/24 Lesotho Demographic and Health Survey.

### Study variables

#### Dependent variable

The outcome variable was self-reported cervical cancer screening uptake, coded as a binary variable, with “1” indicating women who reported ever having undergone cervical cancer screening and “0” indicating those who reported never being screened.

#### Independent variables

Independent variables included individual-level factors such as age, marital status, education level, current employment, wealth index, health insurance, awareness of antiretroviral therapy (ART), current use of ART, history of cancer, receipt of cancer treatment, presence of chronic diseases, receipt of treatment for chronic diseases, history of breast cancer examination, age at first sexual intercourse, number of children, contraceptive use, sexual partnerships, awareness of sexually transmitted infections (STIs), self-reported STI, history of genital sore, and genital discharge. Community-level factors such as place of residence, distance to health facility, mobile telephone use, and media usage were also analyzed.

### Operational definition and categorization

As presented in Table 1, independent variables with binary responses were analyzed as originally coded in the DHS dataset, whereas selected variables that required grouping or transformation for analysis were recoded into meaningful categories for analysis. These included marital status, educational level, wealth index, age at first sexual intercourse, number of children, contraceptive methods, sexual partnerships, and media usage.

**Table 1:** Operationalization and categorization of non-binary independent variables.

Variables	Categorization/operationalization
Marital status	Categorized as married (married and living with a partner) and unmarried (never in union, widowed, divorced, and separated)
Educational level	Categorized as low (no/primary) and high (secondary or higher) education
Wealth index	Categorized as poor (poorest/poorer), middle, and rich (richer/richest)
Age at first sex	Categorized as early (< 15 years), typical (15–19 years), and later (≥ 20 years)
Number of children	Categorized as nulliparous (0), low parity (1–2), and high parity (≥ 3)
Contraceptive methods	Categorized as not using hormonal methods (pill, injections, implants/Norplant, emergency contraception, lactational amenorrhea) and non-hormonal or barrier methods (intrauterine device, male and female condoms, female sterilization, periodic abstinence, withdrawal, other traditional, and other modern methods)
Sexual partnerships	Categorized as no extra-marital sexual partnerships (0–1 partner) and extra-marital sexual partnerships (≥ 2 partners)
Media usage	Categorized as low (< 50% of women exposed) and high (≥ 50% of women exposed). Although the DHS collects individual-level data on radio, television, and newspaper use (yes/no), this information was aggregated at the cluster level to create a community-level variable, based on the proportion of women exposed to at least one type of mass media

### Data analysis

Data cleaning, recoding, and analysis were performed with STATA version 14.0. Descriptive statistics were utilized to describe the sociodemographic, behavioral, and health-related factors of the study population. A two-level mixed-effects logistic regression model was fitted, with individual-level variables treated as fixed effects and cluster-level random intercepts included to account for unobserved community-level heterogeneity. Sampling weights (v005/1,000,000) were applied so that frequencies, percentages, and regression estimates are representative of the national population.

The analysis involved building several models (Model 0, Model I, Model II, and Model III): the null model to assess the variability in cervical cancer screening, a Model I with only individual-level variables, a Model II with only community-level variables, and a Model III that included both levels of variables. Adjusted odds ratios (AORs) with 95% confidence intervals were calculated to identify significant predictors of cervical cancer screening. Measures of random effects, including the intraclass correlation coefficient (ICC), median odds ratio (MOR), and proportional change in variance (PCV), were reported. Model fit was assessed using log-likelihood ratio tests and deviance statistics.

## Result

### Socio-demographic, health, and behavioral characteristics of respondents

According to Table 2, participants were predominantly aged 40–44 years (25.79%), and more than half of the respondents were married (58.82%). Regarding education, the largest proportion of participants had completed secondary education (47.23%). The majority was employed (52.36%) and belonged to the rich wealth index category (49.29%).

**Table 2:** Socio-demographic, health, and behavioral characteristics of respondents in the study.

Variable	Categorized	Unweighted frequency	Weighted frequency
Age	25–29	75 (12.38%)	64.52 (10.56%)
	30–34	108 (17.82%)	111.12 (18.18%)
	35–39	136 (22.44%)	135.58 (22.18%)
	40–44	154 (25.41%)	157.65 (25.79%)
	45–49	133 (21.95%)	142.28 (23.28%)
Marital status	Married	330 (54.46%)	359.48 (58.82%)
	Unmarried	276 (45.54%)	251.68 (41.18%)
Educational level	No education	4 (0.66%)	4.48 (0.73%)
	Primary	284 (46.86%)	257.01 (42.05%)
	Secondary	264 (43.56%)	288.64 (47.23%)
	Higher	54 (8.91%)	61.04 (9.99%)
Current employment	No	318 (52.48%)	291.15 (47.64%)
	Yes	288 (47.52%)	320.02 (52.36%)
Wealth index	Poor	261 (43.00%)	183.05 (29.95%)
	Middle	124 (20.43%)	126.87 (20.76%)
	Rich	221 (36.57%)	301.25 (49.29%)
Health insurance	No	590 (97.36%)	597.55 (97.77%)
	Yes	16 (2.64%)	13.62 (2.23%)
Place of residence	Urban	241 (39.77%)	297.42 (48.66%)
	Rural	365 (60.23%)	313.74 (51.34%)
Distance to a health facility	Big problem	204 (33.66%)	166.24 (27.20%)
	Not a big problem	402 (66.34%)	444.92 (72.80%)
Heard of ARVs to treat HIV?	No	4 (0.66%)	2.38 (0.39%)
	Yes	602 (99.34%)	608.78 (99.61%)
Currently taking ARVs	No	6 (0.99%)	8.19 (1.34%)
	Yes	600 (99.01%)	602.97 (98.66%)
Have cancer or a tumor	No	595 (98.18%)	600.65 (98.28%)
	Yes	11 (1.82%)	10.52 (1.72%)
Receive any treatment for cancer/tumor?	No	6 (54.55%)	6.04 (57.39%)
	Yes	5 (45.45%)	4.48 (42.61%)
Other chronic diseases	No	507 (83.66%)	518.61 (84.86%)
	Yes	99 (16.34%)	92.55 (15.14%)
Receive treatment for chronic diseases	No	13 (13.13%)	18.48 (19.97%)
	Yes	86 (86.87)	74.07 (80.03%)
Breasts examined for cancer by health care provider	No	406 (67.00%)	386.66 (63.27%)
	Yes	200 (33.00%)	224.50 (36.73%)
Age at first sex	Early initiation	54 (8.91%)	47.27 (7.74%)
	Typical initiation	440 (72.61%)	431.14 (70.54%)
	Later initiation	112 (18.48%)	132.75 (21.72%)
Number of children	Nulliparous	40 (6.60%)	47.81 (7.82%)
	Low parity	271 (44.72%)	290.89 (47.60%)
	High parity	295 (48.68%)	272.46 (44.58%)
Contraceptive methods	Not using	212 (34.98%)	213.59 (34.95%)
	Hormonal	248 (40.92%)	222.98 (36.48%)
	Non-hormonal	146 (24.09%)	174.59 (28.57%)
Sexual partnerships	No extramarital sexual partnerships	316 (52.15%)	346.52 (56.70%)
	Extramarital sexual partnerships	290 (47.85%)	264.64 (43.30%)
Used a method of sexual intercourse	No	132 (25.10%)	127.71 (24.08%)
	Yes	394 (74.90%)	402.69 (75.92%)
Ever heard of an STI?	No	268 (44.22%)	234.76 (38.41%)
	Yes	338 (55.78%)	376.41 (61.59%)
Had an STI in the last 12 months	No	575 (94.88%)	571.07 (93.44%)
	Yes	31 (5.12%)	40.09 (6.56%)
Had a genital sore/ulcer in the last 12 months	No	552 (91.09%)	557.71 (90.93%)
	Yes	54 (8.91%)	55.45 (9.07%)
Had a genital discharge in the last 12 months	No	491 (81.02%)	489.66 (80.12%)
	Yes	115 (18.98%)	121.50 (19.88%)
Mobile telephone use	No	42 (6.93%)	38.29 (6.27%)
	Yes	564 (93.07%)	572.87 (93.73)
Media usage (access to radio & TV)	Low	462 (76.24%)	460.14 (75.29%)
	High	144 (23.76%)	151.03 (24.71%)

Most participants were rural residents (51.34%), with 97.77% did not have health insurance. A significant proportion of participants reported that distance to a health facility was not a big problem (72.80%) and that they had access to radio and TV (24.71%). Additionally, almost all respondents had heard of antiretroviral (ARV) treatment for HIV (99.61%) and were taking ARVs (98.66%). The vast majority did not have cancer or a tumor (98.28%) and did not have other chronic diseases (84.86%).

Regarding reproductive health, 70.54% of participants had initiated sexual activity between the ages of 15–19 years. Majorities had low parity (1–2 children) (47.60%) and predominantly used hormonal contraceptives (36.48%). In terms of sexual behavior, most participants reported having had only one sexual partner or no extramarital sexual partner in the last 12 months (56.70%) and indicated that they had used a method during sexual intercourse (75.92%).

More than half of the participants were aware of sexually transmitted infections (STIs), with 61.59% being aware of STIs. STI incidence in the last 12 months was reported as 6.56%, 9.07% reported genital sores or ulcers, and 19.88% reported genital discharge.

### Prevalence of cervical cancer screening among WLHIV

**Fig. 2:**
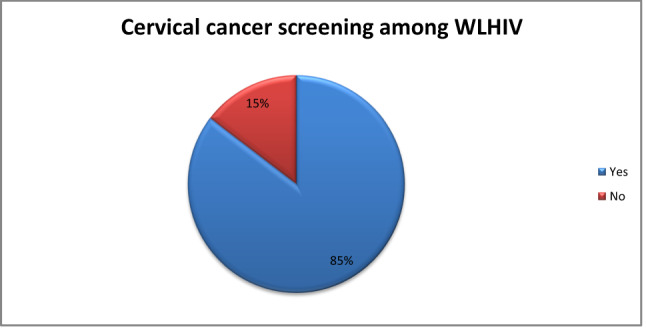
Cervical cancer screening among women living with HIV in Lesotho, 2023/24.

**Fig. 3:**
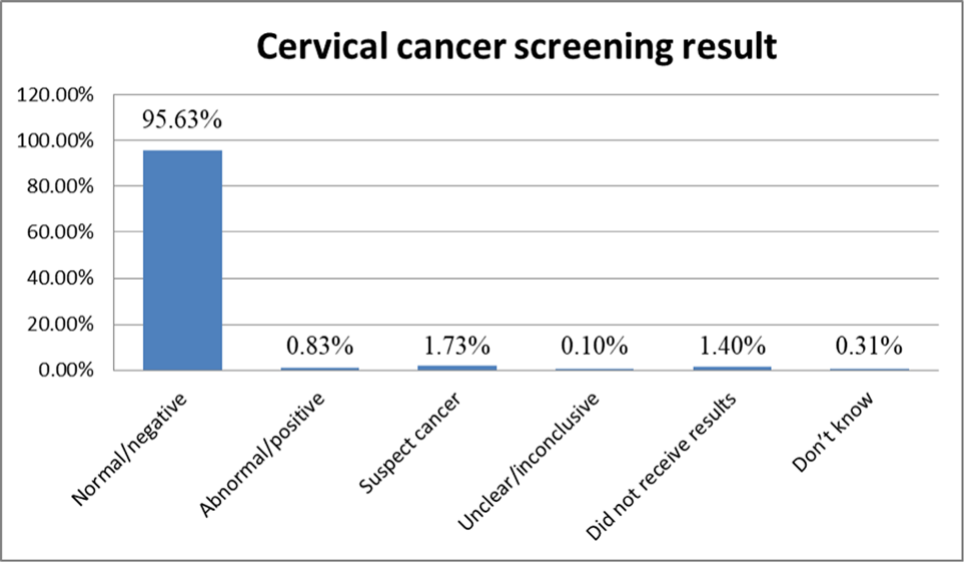
Last cervical cancer screening result among women living with HIV in Lesotho, 2023/24.

The overall cervical cancer screening among women living with HIV was 85.4% [95% CI 82.35–87.98%] (Fig. 2). Among those screened, the majority had normal/negative results (95.63%). The prevalence of abnormal/positive results was 0.83%, 1.73% were classified as suspect cancer, 0.10% were unclear/inconclusive, 1.40% did not receive results, and 0.31% did not know their result (Fig. 3).

### Multilevel mixed-effect logistic regression analysis of cervical cancer screening among WLHIV

As shown in Table 3, the null model showed substantial community-level clustering in cervical cancer screening (variance = 2.38; ICC = 0.42; MOR 4.33), indicating that 42% of the total variance was attributable to differences between communities. Adding individual-level variables (Model I) and community-level variables (Model II) reduced the unexplained variance (PCV = 79.4% and 97.1%, respectively), while the full model (Model III) retained moderate residual variance (0.37; ICC = 0.10; MOR 1.78) and explained 84.4% of the between-community variability. Model III also had the lowest deviance (437.06) and highest log-likelihood (− 218.53), indicating the best fit. Therefore, Model III, which includes both individual- and community-level factors, was selected as the optimal model for identifying predictors of cervical cancer screening among women living with HIV.

**Table 3:** Multivariable mixed-effect logistic regression analysis results of both individual-level and community-level factors.

Variable	Categorized	Null model	Model I AOR (95% CI)	Model II AOR (95% CI)	Model III AOR (95% CI)
Age	25–29		1.00	–	1.00
	30–34		1.91 [0.73–4.95]	–	2.01 [0.78–5.20]
	35–39		1.75 [0.72–4.25]	–	1.79 [0.74–4.34]
	40–44		4.06 [1.51–10.90]	–	4.14 [1.53–11.18]*
	45–49		2.39 [0.90–6.36]		2.43 [0.91–6.51]
Marital status	Married		1.00	–	1.00
	Unmarried		0.79 [0.42–1.47]		0.83 [0.45–1.56]
Educational level	Low-level education		1.00	–	1.00
	High-level education		1.28 [0.69–2.37]	–	1.33 [0.72–2.46]
Currently working	No		1.00		1.00
	Yes		1.01 [0.58–1.74]		0.95 [0.55–1.64]
Wealth index	Poor		1.00	–	1.00
	Middle		1.69 [0.77–3.75]	–	1.35 [0.59–3.05]
	Rich		1.04 [0.54–2.02]		0.60 [0.27–1.32]
Health insurance	No		1.00	–	1.00
	Yes		0.83 [0.12–5.84]		0.74 [0.10–5.19]
Other chronic diseases	No		1.00	–	1.00
	Yes		0.86 [0.42–1.79]		0.87 [0.42–1.80]
Breasts examined for cancer by health care provider	No		1.00	–	1.00
	Yes		5.92 [2.72–12.91]		5.53 [2.54–12.05]*
Age at initial sex	Early initiation		1.00	–	1.00
	Typical initiation		1.26 [0.47–3.39]	–	1.28 [0.47–3.46]
	Later initiation		1.35 [0.44–4.20]		1.46 [0.47–4.53]
Number of children	No children		1.00	–	1.00
	Low parity		0.19 [0.05–0.80]	–	0.19 [0.05–0.78]*
	High parity		0.24 [0.06–1.05]		0.24 [0.06–1.05]
Contraceptive methods	Not using		1.00		1.00
	Hormonal		1.56 [0.80–3.03]	–	1.51 [0.77–2.93]
	Non-hormonal		1.38 [0.69–2.74]	–	1.27 [0.64–2.53]
Sexual partnerships	No extramarital sexual partnerships		1.00	–	1.00
	Extramarital sexual partnerships		1.30 [0.71–2.37]	–	1.26 [0.69–2.31]
Ever heard of an STI	No		1.00	–	1.00
	Yes		1.66 [0.91–3.04]		1.66 [0.91–3.03]
Had an STI in the last 12 months	No		1.00	–	1.00
	Yes		2.53 [0.49–13.13]		2.46 [0.48–12.64]
Had a genital sore/ulcer in the last 12 months	No		1.00	–	1.00
	Yes		1.23 [0.45–3.39]		1.22 [0.44–3.38]
Had genital discharge in the last 12 months	No		1.00	–	1.00
	Yes		0.77 [0.38–1.58]		0.74 [0.36–1.51]
Place of residence	Urban		–	1.00	1.00
	Rural			0.54 [0.32–0.91]	0.50 [0.25–0.99]*
Distance to a health facility	Big problem		–	1.00	1.00
	Not a big problem			1.50 [0.89–2.54]	1.42 [0.77–2.65]
Mobile telephone use	No		–	1.00	1.00
	Yes			1.53 [0.65–3.56]	1.24 [0.45–3.41]
Media usage	Low access		–	1.00	1.00
	High access			1.13 [0.65–1.98]	1.15 [0.59–2.27]
Random effect and model comparisons					
Random effect	Variance	2.38	0.49	0.07	0.37
	ICC	0.42	0.13	0.02	0.10
	MOR	4.33	1.95	1.28	1.78
	PCV	Reff	79.4%	97.1%	84.4%
Model comparison	Log-likelihood ratio	− 253.95	− 221.92	− 247.23	− 218.53
	Deviance	507.90	443.84	494.46	437.06

“*” indicates variables with *p* < 0.05, considered statistically significant.

In the multilevel logistic regression analysis, women aged 40–44 years were significantly more likely to undergo cervical cancer screening (AOR 4.14; 95% CI [1.53–11.18]) compared with women aged 25–29 years. Women who had previously undergone a breast examination by a medical professional had higher odds of cervical cancer screening (AOR 5.53; 95% CI [2.54–12.05]) than those who had not. In contrast, women with low parity were less likely to undergo cervical cancer screening (AOR 0.19; 95% CI [0.05–0.78]) compared with women with no children. Rural residence was associated with a reduced likelihood of cervical cancer screening (AOR 0.50; 95% CI [0.25–0.99]) compared with urban residence.

## Discussion

This study aimed to assess the prevalence and determinants of cervical cancer screening among Lesotho’s HIV-positive women using DHS 2023/24 data. Cervical cancer screening coverage was high, with more than four-fifths of participants reported to have been screened. Older age (40–44 years) and prior breast examination by a medical professional were positively associated with screening uptake, whereas low parity and rural residence were associated with lower odds of screening.

The findings of this research revealed that cervical cancer screening among women living with HIV, was 85.4% (95% CI 82.35–87.98%), which is in line with findings from the USA (83%)^17^ and England (85.7%)^18^. This rate is significantly higher than studies from other regions, including Spain (50.6%)^19^, India (41.15%)^20^, Uganda (44%)^21^, and Ethiopia (27.8%)^22^, but lower than studies in Italy (91%)^23^. The relatively high screening coverage in Lesotho may reflect the integration of cervical cancer screening into HIV care services and the support of donor-funded programs that promote routine screening among women living with HIV. Additionally, ongoing national HPV vaccination efforts may have increased awareness of cervical cancer and preventive behaviors, indirectly encouraging higher screening uptake. The observed high prevalence may also be influenced by self-reported data and is specific to the HIV-positive study population; studies in the general population, including both women living with HIV and those without HIV, generally report lower screening coverage.

In this study, age emerged as a significant determinant; women aged 40–44 were more than four times more likely to undergo screening than women aged 25–29 years. This finding is consistent with studies from Ethiopia^24^, Kenya^25^, and a global analysis comparing LMICs and high-income countries^26^. The possible explanation is that older women tend to have had prior exposure to health care, more exposure to health education campaigns, or family or individual illness experiences^27,28^.

One of the significant findings was that women who had undergone a breast examination by a healthcare provider were over five times more likely to have been screened for cervical cancer than women who had no prior breast examination. This is in line with findings that using other preventive care services raises the uptake of cervical cancer screening, as reported in South Africa^29^ and Malawi^30^ studies. This association indicates that women who use other preventive health services, such as breast examinations, tend to undergo cervical cancer screening. This observation emphasizes a powerful implication for integrated care policy: exposure to one preventive service may encourage uptake of others, reinforcing the benefit of co-locating or linking breast and cervical cancer services^31^.

Another finding of this study was that parity influenced cervical cancer screening uptake; women with low parity (1–2 children) were less likely to undergo cervical cancer screening compared with women with no children. The plausible explanation is that women without children may be more likely to be engaged in general health checkups, family planning, or HIV care services where screening opportunities arise, whereas women with one or two children might have completed initial reproductive health visits and are less consistently engaged in follow-up screening. Similar patterns have been observed in studies conducted in Jamaica^32^, and Ethiopia^33^. In addition, low-parity women may perceive themselves at lower risk of reproductive health issues compared with women without children, or may face competing caregiving responsibilities that reduce opportunities for preventive care^27^.

Furthermore, the study identified rural residence as a significant barrier to cervical cancer screening compared to women living in urban areas, with women living in rural areas being less likely to be screened despite the presence of Lesotho’s mobile outreach programs. This finding is consistent with other studies in Uganda^34^, Nigeria^35^, and Kenya^25^ where rural women face numerous barriers to accessing healthcare. This may reflect challenges such as limited frequency of mobile clinic visits, low community awareness, and logistical barriers in accessing screening services. To strengthen rural coverage, lessons can be drawn from neighboring countries like Rwanda’s HPV-DNA testing model and Malawi’s integration of VIA screening into routine health services, which have demonstrated increased uptake in rural populations^36,37^.

Overall, the findings highlight the need for targeted strategies to promote equitable access to cervical cancer screening in Lesotho. Integrating screening into routine HIV care and other preventive services could enhance uptake by utilizing women’s existing contact with the health system. Expanding services to rural and underserved areas through mobile outreach, community mobilization, and strengthened clinic–community linkages remains crucial. Tailored health education approaches, including those directed at low-parity women, may further address gaps in utilization. Promoting multiple preventive health behaviors within community health platforms may also contribute to improved screening coverage. Furthermore, future studies should consider district-, sub-district-, or community-level analyses to identify geographic disparities and guide targeted interventions.

This study highlights several strengths in its approach to investigating cervical cancer screening among women living with HIV. It employs a recent nationally representative dataset and applies multilevel mixed-effects modeling analysis, enabling a comprehensive assessment of both individual and community-level factors influencing screening behaviors. By concentrating on a high-risk, disadvantaged population, the study contributes valuable insights that can inform global strategies for cervical cancer control. However, the study also presents notable limitations, particularly its cross-sectional design, which impedes the ability to draw causal conclusions. Furthermore, essential psychosocial factors such as knowledge, stigma, and screening motivation were not considered, potentially impacting screening behaviors. There is also a concern regarding the reliability of self-reported screening status due to possible social desirability bias, and pertinent data on the frequency and recency of screenings were not collected.

## Conclusion

Cervical cancer screening uptake among women living with HIV in Lesotho appears encouraging; however, important disparities persist. Screening behavior is influenced by demographic, reproductive, healthcare access, and residence-related factors, indicating that not all women benefit equally from existing screening efforts. Strengthening integrated, equitable, and context-specific screening strategies within HIV care services may contribute to further improving coverage and reducing cervical cancer–related morbidity and mortality in this high-risk population.

## Data Availability

Data are available online from a public, open-access repository https://dhsprogram.com/data.

## Abbreviations

AOR: Adjusted odds ratio
COR: Crude odds ratio
DHSs: Demographic and Health Surveys
EAs: Enumeration areas
HPV: Human papillomavirus
ICC: Intra-cluster correlation coefficient
IR: Individual women’s record
LMICs: Low- and middle-income countries
MOR: Median odds ratio
PCV: Proportional change in variance
STI: Sexually transmitted infection
WLHIV: Women living with HIV
